# Combined adipose-derived mesenchymal stem cell and antibiotic therapy can effectively treat periprosthetic joint infection in rats

**DOI:** 10.1038/s41598-023-30087-z

**Published:** 2023-03-09

**Authors:** Yuki Yamamuro, Tamon Kabata, Takayuki Nojima, Katsuhiro Hayashi, Masaharu Tokoro, Yoshitomo Kajino, Daisuke Inoue, Takaaki Ohmori, Junya Yoshitani, Takuro Ueno, Ken Ueoka, Atsushi Taninaka, Tomoyuki Kataoka, Yoshitomo Saiki, Yu Yanagi, Hiroyuki Tsuchiya

**Affiliations:** 1 Department of Orthopaedic Surgery, Graduate School of Medical Sciences, Kanazawa University, 13-1 Takaramachi, Kanazawa, Ishikawa 920-8641 Japan; 2 Department of Pathology and Laboratory Medicine, Kanazawa University, 13-1 Takaramachi, Kanazawa, Ishikawa 920-8641 Japan; 3 Department of Global Infectious Diseases, Graduate School of Medical Sciences, Kanazawa University, 13-1 Takaramachi, Kanazawa, Ishikawa 920-8641 Japan

**Keywords:** Stem cells, Medical research

## Abstract

Periprosthetic joint infection (PJI) is characterized by biofilm infection, which is difficult to alleviate while preserving implant integrity. Furthermore, long-term antibiotic therapy may increase the prevalence of drug-resistant bacterial strains, necessitating a non-antibacterial approach. Adipose-derived stem cells (ADSCs) exert antibacterial effects; however, their efficacy in PJI remains unclear. This study investigates the efficacy of combined intravenous ADSCs and antibiotic therapy in comparison to antibiotic monotherapy in a methicillin-sensitive Staphylococcus aureus (MSSA)-infected PJI rat model. The rats were randomly assigned and equally divided into 3 groups: no-treatment group, antibiotic group, ADSCs with antibiotic group. The ADSCs with antibiotic group exhibited the fastest recovery from weight loss, with lower bacterial counts (p = 0.013 vs. no-treatment group; p = 0.024 vs. antibiotic group) and less bone density loss around the implants (p = 0.015 vs. no-treatment group; p = 0.025 vs. antibiotic group). The modified Rissing score was used to evaluate localized infection on postoperative day 14 and was the lowest in the ADSCs with antibiotic group; however, no significant difference was observed between the antibiotic group and ADSCs with antibiotic group (p < 0.001 vs. no-treatment group; p = 0.359 vs. antibiotic group). Histological analysis revealed a clear, thin, and continuous bony envelope, a homogeneous bone marrow, and a defined, normal interface in the ADSCs with antibiotic group. Moreover, the expression of cathelicidin expression was significantly higher (p = 0.002 vs. no-treatment group; p = 0.049 vs. antibiotic group), whereas that of tumor necrosis factor (TNF)-α and interleukin(IL)-6 was lower in the ADSCs with antibiotic group than in the no-treatment group (TNF-α, p = 0.010 vs. no-treatment group; IL-6, p = 0.010 vs. no-treatment group). Thus, the combined intravenous ADSCs and antibiotic therapy induced a stronger antibacterial effect than antibiotic monotherapy in a MSSA-infected PJI rat model. This strong antibacterial effect may be related to the increased cathelicidin expression and decreased inflammatory cytokine expression at the site of infection.

## Introduction

Periprosthetic joint infection (PJI), an implant-related infection, is one of the most serious complications that can occur following total joint arthroplasty. PJI is characterized by biofilm infections, wherein biofilm-forming bacteria escape the host’s immune response and become resistant to antibiotics^1^. Moreover, prolonging antibiotic therapy to quell infection while preserving the integrity of the implant is extremely difficult, and revision surgeries are often required^2^. Furthermore, with an increase in the number of arthroplasty procedures being performed, an increase in associated infections will also inevitably occur, posing a significant threat to society^3^. In clinical practice, removing implants might be impossible as this process can negatively impact quality of life, and the development of bacterial resistance during long-term antibiotic therapy is also a concern^4^. Therefore, a non- antibiotics strategy is needed to prevent biofilm infection while preserving implant integrity.

Previous studies have focused on strategies that activate and modulate the immune system^5,6^ or induce the release of antimicrobial peptides from mesenchymal stem cells (MSCs)^7,8^. MSCs have been studied in various fields and have exhibited therapeutic efficacy against coronavirus disease 2019 infection, sepsis, and pneumonia^9^. Additionally, MSCs, including adipose-derived stem cells (ADSCs), may be effective for treating biofilm infections and infection-related sepsis when administered intravenously in combination with antibiotics^10,11^. However, this therapy has not yet been evaluated in detail for PJI. Furthermore, the antibacterial efficacy of the combined intravenous ADSCs and antibiotic therapy, as well as their effect on host physiology, including bone and soft tissue structure, remain unknown. Therefore, in the current study, we examined whether combined ADSCs and antibiotic therapy is superior to antibiotic monotherapy in a methicillin-sensitive *Staphylococcus aureus* (MSSA)-infected PJI rat model.

## Results

### Infection model and systemic response

**Figure 1:**
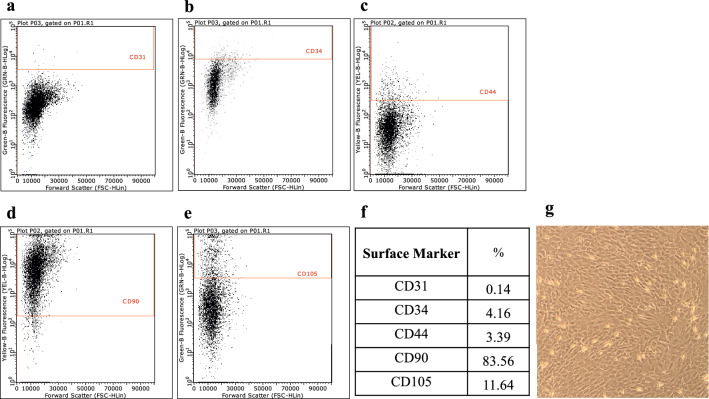
Flow cytometric analysis. (**a‒e**) Flow cytometric analysis after cell culturing. (**f**) Percentage of ADSCs in each sub-population. (**g**) Spindle-shaped cells were observed in the culture plate, a typical appearance of mesenchymal stem cells. *ADSC* adipose-derived mesenchymal stem cell.

**Figure 2:**
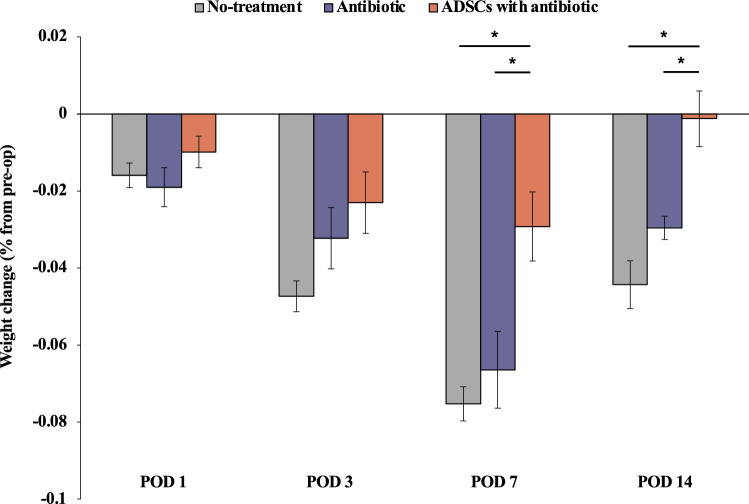
Evaluation of weight change. Weight percent change from pre-operation day 1 baseline values. Weight change was significantly smaller in the ADSCs with antibiotic group than in the other groups on POD7 and POD14. All statistical analyses were performed using Welch ANOVA followed by Tukey HSD post-test (n = 6 rats per group). The error bars are defined as standard error of the mean. *p < 0.05. *ADSC* adipose-derived mesenchymal stem cell.

Fidelity of the ADSCs was confirmed using flow cytometry. Flow cytometric analysis showed that the subgroup of CD90+ ADSCs had the highest population. Moreover, the cells were spindle-shaped, a typical morphological feature of ADSCs (Fig. 1). All rats survived and were active during the observation period. The systemic response to infection was indirectly quantified by measuring changes in body weight. Rats in all three groups lost weight initially after surgery, reaching the lowest point on postoperative day (POD) 7. However, the weight change was significantly smaller in the ADSCs with antibiotic group than in the other groups on POD7 (Welch ANOVA followed by Tukey HSD post-test, p = 0.003 vs. no-treatment group; p = 0.015 vs. antibiotic group) and POD14 (Welch ANOVA followed by Tukey HSD post-test, p < 0.001 vs. no-treatment group; p = 0.009 vs. antibiotic group). Notably, the weight in the ADSCs with antibiotic group recovered to baseline on POD 14 (Fig. 2).

### Local response

**Figure 3:**
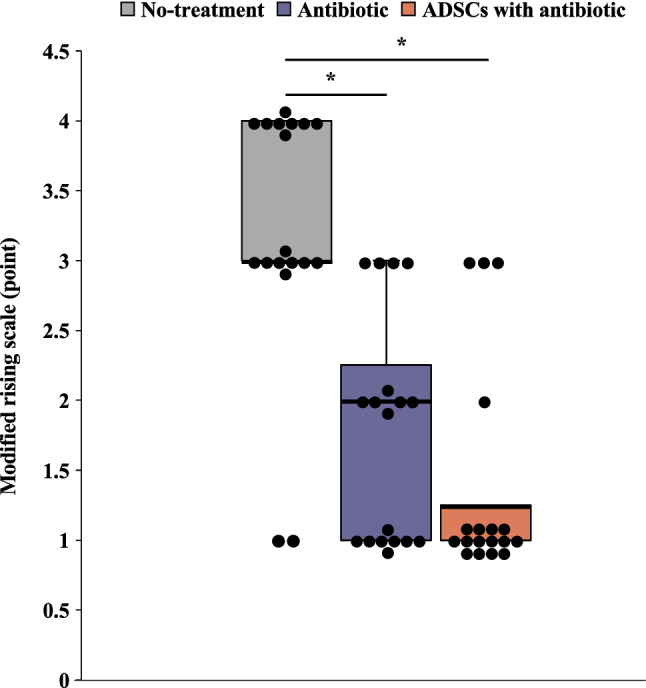
Evaluation of the modified Rising scale. All statistical analyses were performed using Welch ANOVA followed by Tukey HSD post-test (n = 6 rats per group). Data are reported as the median ± interquartile range. *p < 0.05. *ADSC* adipose-derived mesenchymal stem cell.

The modified Rissing score was used to evaluate localized infection on POD14. This score was the lowest in the ADSCs with antibiotic group; however, no significant difference was observed between the antibiotic group and ADSCs with antibiotic group (Welch ANOVA followed by Tukey HSD post-test, p < 0.001 vs. no-treatment group; p = 0.359 vs. antibiotic group; Fig. 3). Assessment of the intra-rater reliability of the modified Rissing score revealed an intra-class coefficient of 0.882 (95% confidence interval, 0.740–0.952).

### Bone quality evaluation by μ-computed tomography (µCT)

**Figure 4:**
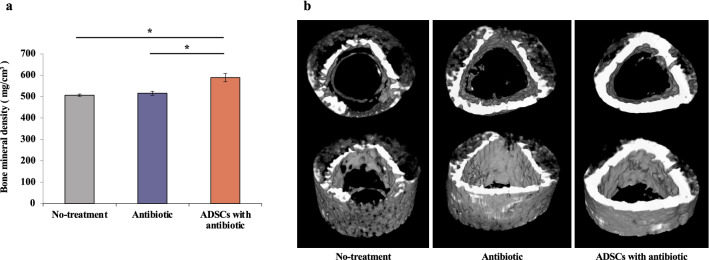
Evaluation of μCT. (**a**) Quantification of the femoral bone mineral density of each femur in the three groups 14 days after insertion of the K-wire. (**b**) Three‐dimensional μCT axial images of the distal femoral region 14 days after insertion of the K-wire. All statistical analyses were performed using Welch ANOVA followed by Tukey HSD post-test (n = 6 rats per group). The error bars are defined as standard error of the mean. *p < 0.05. *ADSC* adipose-derived mesenchymal stem cell.

*Ex vivo* μCT analysis on POD14 showed that the mean periprosthetic bone mineral density (BMD) was 506.6 ± 12.7 mg/cm^3^ (95% confidence interval 95% CI 493.3–520.0) in the no-treatment group, 516.2 ± 23.1 mg/cm^3^ (95% CI 491.9–540.5) in the antibiotic group, and 589.2 ± 46.8 mg/cm^3^ (95% CI 540.0–638.3) in the ADSCs with antibiotic group. The ADSCs with antibiotic group exhibited the highest BMD (Welch ANOVA followed by Tukey HSD post-test, p = 0.015 vs. no-treatment group; p = 0.025 vs. antibiotic group; Fig. 4a).

The three-dimensional (3D) rendering and qualitative evaluation of the μCT images revealed a clear difference in bone quality among the three groups. The 3D μCT axial slice images showed osteolysis and an increased bone size associated with periosteal reaction, which was clearly observed in the distal region of the infected femurs in the no-treatment group and antibiotic group (Fig. 4b).

### Culture-based quantification of bacteria on the implant

**Figure 5:**
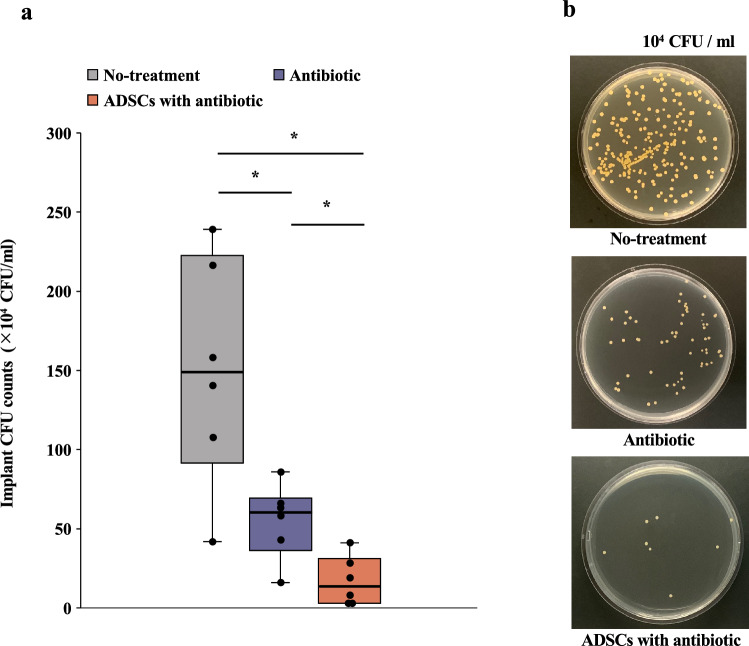
CFU assay. (**a**) Antibacterial activity determined using the spread plate method for implants. (**b**) Photograph of a tryptic soy broth agar plate of each group. All statistical analyses were performed using Welch ANOVA followed by Tukey HSD post-test (n = 6 rats per group). Data are reported as the median ± interquartile range. *p < 0.05. *CFU* colony-forming unit, *ADSC* adipose-derived mesenchymal stem cell.

*Ex vivo* colony-forming units (CFUs) from sonicated Kirschner wires (K-wires) were enumerated on POD14. The implants from the no-treatment group had a mean CFU count of 150.7 ± 72.1 (95% CI 75.0–226.3) × 10^4^ CFU/mL; those from the antibiotic group had a mean of 55.0 ± 72.1 (95% CI 30.3–79.7) × 10^4^ CFU/mL; and from the ADSCs with antibiotic group had a mean of 17.0 ± 15.3 (95% CI 0.9–33.1) × 10^4^ CFU/mL. Although bacteria were detected in the ADSCs with antibiotic group, the bacterial burden was significantly lower than that in the no-treatment or antibiotic groups (Welch ANOVA followed by Tukey HSD post-test, p = 0.013 vs. no-treatment group; p = 0.024 vs. antibiotic group; Fig. 5a and b).

### Histological analysis

**Figure 6:**
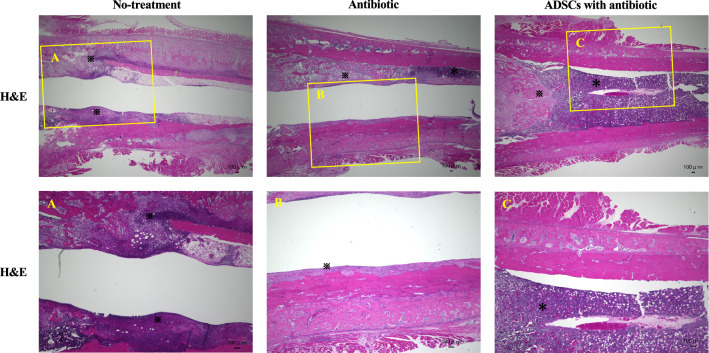
Histologic analysis. Representative photomicrographs of histologic sections (1 of the 6 rats per group, with similar results). Low and high magnification of H&E-stained joint specimens. The H&E-stained histology sections taken from the transverse plane across the implant revealed a clear, thin, and continuous bony envelope and homogeneous bone marrow as well as a defined normal interface between these structures in the ADSCs with antibiotic group. (**A**), (**B**), and (**C**) represent high-magnification images of the boxed regions. ^※^abscess formation, *bone marrow. *H&E* hematoxylin and eosin, *ADSC* adipose-derived mesenchymal stem cell.

To determine the microscopic location of the inflammatory infiltrate and bacterial inoculum around the implant, histological sections of the distal femur were evaluated from the three groups at the end of the experiment on POD14. The hematoxylin and eosin-stained sections collected from the transverse plane across the implant revealed a clear, thin, and continuous bony envelope and homogeneous bone marrow and a defined normal interface between these structures in the ADSCs with antibiotic group. The antibiotic group showed a clear, thick, and continuous bony envelope; however, the bone marrow became less cellular, and the interface was not well-defined. In contrast, abscess formation and gradual disruption of bone integrity with severe discontinuity were observed in the no-treatment group (Fig. 6).

### Gene expression levels of rat cathelicidin-related antimicrobial peptide (rCRAMP), tumor necrosis factor(TNF)-α, interleukin (IL-6), IL-1β, and glyceraldehyde 3-phosphate dehydrogenase (GAPDH)

**Figure 7:**
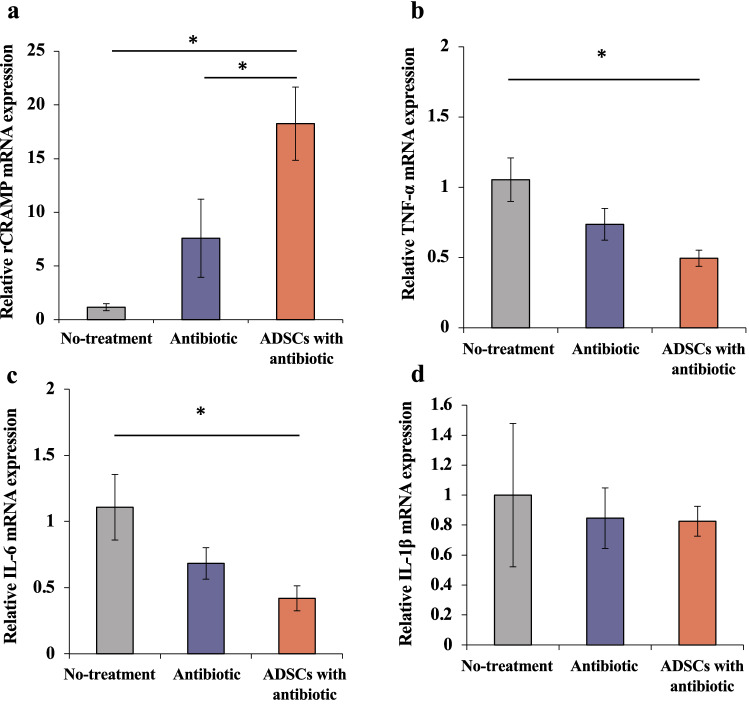
Real-time reverse transcription–polymerase chain reaction (RT-PCR). (**a-d**) Gene expression of rCRAMP,TNF-α, IL-6, and IL-1β in the three groups. All statistical analyses were performed using Welch ANOVA followed by Tukey HSD post-test (n = 6 rats per group). The error bars are defined as standard error of the mean. *p < 0.05. *mRNA* messenger ribonucleic acid, *rCRAMP* rat cathelicidin-related antimicrobial peptide, *TNF* tumor necrosis factor, *IL* interleukin, *ADSC* adipose-derived mesenchymal stem cell.

The control GAPDH gene was stably expressed at the site of infection in all groups. Reverse transcription Polymerase Chain Reaction (RT-PCR) revealed a significant increase in the expression of the rCRAMP gene in the ADSCs with antibiotic group (Welch ANOVA followed by Tukey HSD post-test, p = 0.002 vs. no-treatment group; p = 0.049 vs. antibiotic group; Fig. 7a). The TNF-α and IL-6 expression in the ADSCs with antibiotic group was lower than that in the no-treatment group but not significantly lower than that in the antibiotic group (Welch ANOVA followed by Tukey HSD post-test, TNF-α, p = 0.010 vs. no-treatment group; IL-6, p = 0.010 vs. no-treatment group; Fig. 7b, c). The IL-1β gene expression did not significantly differ among the three groups (Fig. 7d).

### DiI labeling studies

**Figure 8:**
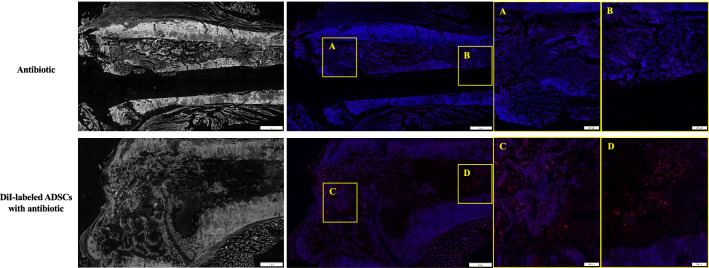
Representative images of DiI labeling at the site of infection. Frozen sections were prepared from rats in the antibiotic group not injected with labelled cells **(**top**)**, and 14 days after transplantation of ADSCs labeled with DiI **(**bottom**)** (1 of the 3 rats per group, with similar results). For identification of tissues following DiI labeling, the gray-scale scale (16 bit) of the DiI-labeled section was used. On POD14, DiI-positive (red) cells were distributed through the trabecular bone around the K-wire. (**A**), (**B**), (**C**), and (**D**) represent high-magnification images of the boxed regions. *ADSC* adipose-derived mesenchymal stem cell.

On POD14, DiI-positive (red) cells were distributed through the trabecular bone around the K-wire (Fig. 8). The observed fluorescence was specific to ADSCs, and was not an experimental artefact or autofluorescence as confirmed by the absence of staining in antibiotic-only-treated samples (Fig. 8).

## Discussion

The combined intravenous ADSCs and antibiotic therapy exhibited good antibacterial effects in the MSSA-infected PJI rat model. Moreover, the combined therapy was superior to antibiotic monotherapy in reducing weight loss, bacterial counts in implant biofilms, and abscess formation. The combined therapy also effectively minimized peri-implant osteolysis and reduced BMD, significantly increasing cathelicidin expression at the site of infection. These results emphasize that ADSCs do not interfere with, but rather enhance, the effects of antibiotic agents and that the underlying mechanism may include increased cathelicidin expression at the site of infection. MSCs, including ADSCs, exert antimicrobial activity through multiple complementary mechanisms of action: indirectly through immunomodulators and directly through the release of antimicrobial peptides^7,12–15^. We selected ADSCs as they are abundant in the subcutaneous adipose tissue and can be readily harvested in clinical settings using syringes or minimally invasive liposuction.

PJI has systemic consequences with negative adverse effects on patient mortality and quality of life^16,17^. The systemic response to infection can be determined by measuring weight change because slow, subtle weight loss represents the earliest reliable sign of a worsening systemic condition^18^. A recent study has shown that intravenous ADSCs act synergistically with antibiotics to reduce organ damage within the urinary system and mortality in a rat model of sepsis in which enteric bacteria were intraperitoneally injected, suggesting that intravenous ADSCs therapy is effective against systemic infections^11^. Similarly, in present study, the combined intravenous ADSCs and antibiotic therapy prevented the exacerbation of systemic conditions caused by PJI. Although no significant difference was observed between the ADSCs with antibiotic group and antibiotic group, the combined intravenous ADSCs and antibiotic therapy suppressed the local infection score and expression of inflammatory cytokines (TNF-α and IL-6) at the site of infection, suggesting an association between ADSCs and an improved antibacterial effect.

PJI causes periarticular osteolysis and periosteal osteogenesis, which may adversely affect clinical outcomes. Established infections worsen bone quality over time, resulting in a decreased BMD and cortical thickening^19,20^. In clinical practice, the reduction of bone stock around PJI is a major problem^21^. Our results suggest that the combined intravenous ADSCs and antibiotic therapy inhibits bone stock reduction around infected implants and facilitates two-stage reconstruction, which is necessary in clinical practice. A previous study has shown that ADSCs regulate B cells, promote osteoblast formation, and inhibit osteoclasts, thereby restoring the regenerative capacity of bone defects after infection^22^. However, even in the intravenous ADSCs with antibiotic group, the femoral BMD was lower than that in normal rats.

Johnson et al. reported a significant antimicrobial effect of intravenous ADSCs in an *in vivo* subcutaneous mesh mouse model, showing a reduction in the number of bacteria in the peri-implant biofilm due to the interaction with antibiotics^10^. In our MSSA-infected PJI rat model, the combined intravenous ADSCs and antibiotic therapy significantly reduced the number of bacteria in the biofilm and enhanced the antibacterial effect compared with the results of antibiotic monotherapy. Furthermore, pathological evaluation revealed that the extent of the abscess was reduced.

Notably, we found that the cathelicidin expression at the site of infection was significantly increased in the ADSCs with antibiotic group. ADSCs and other MSCs secrete various antimicrobial peptides, including cathelicidin^8,15^. Bacteria are less likely to develop resistance to antimicrobial peptides than to antibiotics; therefore, antimicrobial peptides have attracted considerable attention in recent years^23^. Cathelicidins comprise a major antimicrobial peptide family in mammals that exerts its killing effect by disrupting bacterial membrane integrity and inhibiting biofilms^24–26^. Thus, cathelicidin at the site of infection may have enhanced the antibacterial effects. Additionally, systemically administered MSCs tend to remain in the lungs; however, they can be distributed to multiple organs and tissues away from the administration site^27^. Particularly, compared to locally administered ADSCs, intravenously administered ADSCs effectively suppress deep-seated bacterial infections by migrating from the lungs to the site of infection over several days, resulting in lower bacterial counts in the wounds^10^. ADSCs then accumulate around implants, where direct administration is difficult, leading to an increase in cathelicidin expression at the site of infection. Therefore, our study findings suggest that the increase in cathelicidin expression at the site of infection is related to the accumulation of ADSCs. Intravenous ADSCs may, therefore, prove effective as a treatment against PJI.

Several limitations were noted in the current study. The study was conducted exclusively in rats; therefore, the results may differ in large mammals, including humans. The PJI rat model was created by inserting K-wires retrogradely, which may not reproduce the actual loading environment of the artificially infected joint. Moreover, our results may differ from those of other studies owing to differences in the expression markers of ADSCs. At present, no specific marker or combination of markers has been identified that specifically defines MSCs. Phenotypically, ex vivo-expanded MSCs express several nonspecific markers, including CD90, CD105, CD73, CD166, CD44, and CD29^28,29^. MSCs are devoid of hematopoietic and endothelial markers, such as CD11b, CD14, CD31, CD34 and CD45^28^. Our ADSCs were consistent with the characteristics of the MSCs. In addition, in our study, ADSCs localization to the site of infection was confirmed by in vivo cell tracking; furthermore, the combined therapy increased the expression of cathelicidin and decreased that of inflammatory cytokines at the site of infection. However, further investigation is needed to elucidate the relationship and mechanism between the antibacterial activity of the combined therapy and the expression of inflammatory cytokines and cathelicidin. Furthermore, the data were collected only after 14 days, whereas results obtained at 3 and 7 days would provide additional information regarding the evolution of the antibacterial effect of ADSCs combined with antibiotic treatment. Therefore, additional studies are required encompassing larger total numbers of animals to permit evaluation at multiple time points. Finally, only the effects of ciprofloxacin were examined; different results may occur using other antibiotics. However, quinolones are excellent antimicrobial agents in terms of their bioavailability, antibacterial activity, and tolerability^30^. Moreover, ciprofloxacin has been extensively tested for the long-term treatment of implant-related staphylococcal infections and can be applied in clinical practice^31^.

In conclusion, the combined intravenous ADSCs and antibiotic therapy induces a stronger antibacterial effect than antibiotic monotherapy in a MSSA-infected PJI rat model, with earlier recovery from weight loss, reduced peri-implant bacterial counts, and reduced peri-implant BMD. This strong antibacterial effect may be related to the increased expression of cathelicidin and decreased expression of inflammatory cytokines at the site of infection. Our results suggest that the combined intravenous ADSCs and antibiotic therapy may be used to treat patients with PJI who show an inadequate response to conventional antibiotic monotherapy.

## Materials and methods

### Bacteria and biofilm formation

MSSA strain ATCC29213 (American Type Culture Collection, Manassas, VA, USA) was used as it tends to form biofilms^32,33^. MSSA was streaked onto plates containing tryptic soy broth and Bacto agar (BD Biosciences, Franklin Lakes, NJ, USA) and grown overnight in 5 mL of tryptic soy broth at 37 °C in a shaking incubator. MSSA cells in the incubation medium were grown to the early exponential growth phase (0.2–0.3 optical density at 600 nm), corresponding to 5.0 × 10^7^ CFU/mL.

### Isolation of ADSCs

Adipose tissue (~1.5 g) was obtained from Wistar rats (female; 12 weeks old; Japan SLC Corp., Shizuoka, Japan). ADSCs were prepared by modifying previously reported methods^34^. Further details can be found in the Supplementary file. Cellular characteristics (i.e. expression of stem cell surface markers) were determined using flow cytometric analysis after labeling ADSCs with appropriate antibodies of cultivation.

### Rat PJI model, surgical procedures, and animal grouping

Wistar rats (female; 12 weeks old; Japan SLC Corp.) were housed under specific pathogen-free conditions with a 12-h light/dark cycle and *ad libitum* access to a certified diet (CRF-1; Oriental Yeast Corp., Tokyo, Japan) and water (chlorine concentration; 10 ppm). The drinking, feeding behavior, and body weight of the rats were monitored regularly. The animals were acclimatized for 7 days before undergoing the implant operation.

Rats were anesthetized with midazolam (2.5 mg/kg; Astellas Pharma, Tokyo, Japan), medetomidine (0.5 mg/kg; Zenoaq, Fukushima, Japan), and butorphanol tartrate (2.5 mg/kg; Meiji Seika Pharma, Tokyo, Japan). To establish infection, A medical-grade K-wire (1.2 mm diameter; Synthes Inc., West Chester, PA, USA) was incubated in an overnight culture with MSSA strain ATCC29213 and then air-dried for 20min prior to insertion. This MSSA strain exposure coats the screw with 5×10^7^ CFU. The K-wire was surgically placed into the distal femur as previously described^34–36^. Briefly, the skin overlying the leg was shaved and cleaned with iodine solution. A medial parapatellar approach was used, and the patella was dislocated laterally to access the knee joint. The femoral medullary canal was reamed with an 18-gauge needle and the K-wire was placed in a retrograde fashion with 1 mm of the wire protruding into the joint space. The quadriceps-patellar complex was reduced to the anatomic position, and the wound was closed with nylon 4-0 sutures. Rats were randomly assigned and equally divided into three groups: no-treatment, antibiotic (ciprofloxacin [3.0 mg/kg per day intravenously]), and ADSCs [5.0 × 10^5^ cells intravenously 30 min, 6 h, and 18 h after the surgical procedure]) with antibiotic (ciprofloxacin [3.0 mg/kg per day intravenously] groups. The ADSC dose, based on a previous report^37^, is considered to not induce adverse effects, including a high mortality rate. Additionally, a previous report showed that a ciprofloxacin dose of 3.0 mg/kg per day caused no adverse effects or unstable conditions in rats^11^. MSSA induced infection in 100% of the untreated rats with no significant differences in the initial body weights between the different groups.

After evaluating the general overall condition and soft tissue swelling, the rats were euthanized on POD 14 using thiopental sodium (100 mg/kg body weight). Tissues from the knee joint space, femur, and implant were harvested in a sterile manner for *ex vivo* analyses.

### Weight monitoring

Weight change (n = 6 rats per group) was calculated as a percentage change based on the preoperative weight to quantitatively measure the systemic response to infection. Preoperative baseline measurements were performed on the day before surgery. The weight of the rats was also evaluated on PODs 1, 3, 7, and 14.

### Local tissue scoring

Soft tissue and bone damage (n = 6 rats per group) on POD14 was scored by three examiners (D.I, A.T. and T.K.) blinded to the rats according to a modified Rissing scoring^38,39^. Further details can be found in Supplementary file.

### µCT

μCT imaging (n = 6 rats per group) was performed on POD14 to determine the degree of infection within the femoral region of interest. Considering that image artifacts from the K-wires may cause artifacts in the reconstructed μCT images, isolated femurs from rats with the wire removed were subjected to μCT scanning (LaTheta LCT-200; Hitachi Aloka Medical, Tokyo, Japan), operating at 50 kV and 0.5 mA (radiation exposure remained below 40 mSv). BMD was calculated automatically using LaTheta software (version 3.51). Reconstructed μCT images were initially visualized in three dimensions (3D) to evaluate changes in bone morphology resulting from implant infection. A threshold-limited 3D rendering was created to visualize bone damage.

### Quantitative evaluation with the spread plate method

Implants were harvested (n = 6 rats per group) from each group. Based on a previous report, the bacterial burden on the implants was determined using a CFU assay^40,41^. To quantify living bacteria adherent to the implant within the biofilm, the removed implants were placed individually into 1.5-mL microtubes containing PBS (1 mL at 4 °C), vortexed for 15 s and sonicated for 5 min at 40 Hz in a water bath (Bransonic 5210; Branson Ultrasonics, Brookfield, CT, USA), followed by an additional 1 min of vortexing. The spread plate method was used to quantitatively evaluate the biofilm; the solution containing each bacterium from the biofilm was serially diluted 10-fold with PBS, followed by culturing on an agar plate at 37 °C for 24 h. MSSA was cultured on tryptic soy broth agar plates. The bacterial CFUs obtained from the implant were determined by counting the CFUs after culturing on plates overnight.

### Histological analysis

At the established endpoint (POD14), the femurs isolated from the rats were fixed in 10% neutralized formalin solution and dehydrated using an ethanol gradient (70%, 80%, 90%, and 100%). The fixed specimens were decalcified in 10% formic sodium citrate solution, embedded in paraffin, and sectioned in the coronal plane. The sections were stained with hematoxylin and eosin, and the slides were observed using an optical microscope (Biorevo BZ-9000; Keyence Corp., Osaka, Japan).

### Real-time RT-PCR

At the established endpoint (POD14), total RNA was extracted from the knee tissue of the rats (n = 6 rats per group). The mRNA expression of rCRAMP, TNF-α, IL-6 and IL-1b was evaluated by quantitative PCR. All values were normalized to the level of the GAPDH gene, and relative gene expression levels were calculated using the 2^−ΔΔCt^ method^42^. Further details can be found in the Supplementary file (Supplementary Table S1).

### DiI labeling studies

Tissue sections were evaluated to determine the location of ADSCs following injection. To confirm the location of the injected ADSCs, they were labeled with the fluorescent dye DiI (Vybrant DiI Cell Labeling Solution; Life Technologies, Carlsbad, CA, USA) before injection. DiI binds to cellular thiols and has long-term stability, enabling the tracing of DiI-labeled transplanted cells in the host tissue. The concentration of ADSCs was adjusted to 5.0 × 10^5^ cells/mL; DiI (5 μL/mL) was dissolved in the cell culture media and incubated for 15 min at 37 °C in a 5% CO_2_ incubator for ADSCs labeling. The filtrate was centrifuged at 180×*g* for 5 min at 25 °C and the supernatant was removed to separate the DiI from the filtrate. The ADSCs were centrifuged twice with Dulbecco’s modified Eagle medium under the same conditions and the supernatant was removed. We used separate rats for this experiment (n = 3 rats per antibiotic group and DiI-labeled ADSCs with antibiotic group). On day 14 post-injection, a frozen section was prepared using Kawamoto’s film method in the sagittal plane^43^. For identification of tissues following DiI labeling, the gray-scale scale (16 bit) of the DiI-labeled section was used.

### Statistical analysis

All continuous variables were assessed for normality using the Shapiro–Wilk test. Normally distributed data were expressed as the mean ± standard error. Data were analyzed using SPSS software (version 25.0; SPSS, Inc., Armonk, NY, USA). Multiple groups were compared using the Welch ANOVA followed by Tukey HSD or Games-Howell post-hoc test. For all analyses, results were considered statistically significant at p < 0.05.

### Ethical review committee statement

The investigational protocol was approved by the Kanazawa University Advanced Science Research Centre (Approval Number: AP-194052), and all animals were treated in accordance with Kanazawa University Animal Experimentation Regulations. The study was carried out in compliance with the ARRIVE guidelines.﻿

## Supplementary Information

Supplementary Information.

## Data Availability

All the data used to draw the conclusions of this paper are available in the data presented in the figures and/or table. The raw/processed data required to reproduce these findings are available from the corresponding author upon request.
