# Supplementary material for: Combined adipose-derived mesenchymal stem cell and antibiotic therapy can effectively treat periprosthetic joint infection in rats
Source: Sci Rep. 2023 Mar 9;13:3949. doi: 10.1038/s41598-023-30087-z (PMC9996572; doi:10.1038/s41598-023-30087-z)
Supplement: Supplementary file 1 — Supplementary Information. [file 41598_2023_30087_MOESM1_ESM.docx]

**Supplementary information**

**Combined Adipose-Derived Mesenchymal Stem Cell and Antibiotic Therapy Can Effectively Treat Periprosthetic Joint Infection in Rats**

Yuki Yamamuro, Tamon Kabata, Takayuki Nojima, Katsuhiro Hayashi, Masaharu Tokoro, Yoshitomo Kajino, Daisuke Inoue, Takaaki Ohmori, Junya Yoshitani, Takuro Ueno, Ken Ueoka, Atsushi Taninaka, Tomoyuki Kataoka, Yoshitomo Saiki, Yu Yanagi, Hiroyuki Tsuchiya

**Supplementary methods**

***Details of isolation of ADSCs***

ADSCs were prepared by modifying previously reported methods [1]. Adipose tissue (~1.5 g) was obtained from Wistar rats (female; 12 weeks old; Japan SLC Corp., Shizuoka, Japan) and immediately washed with 200 mL of phosphate-buffered saline (PBS; Wako Pure Chemical Corp., Osaka, Japan). The tissue was cut into strips using sharp sterile surgical scissors within 5 min. Collagenase (Wako Pure Chemical Corp.) was solubilized in PBS to 0.12% final concentration in 20 mL and used to dissolve adipose tissue during a 45-min incubation in a 37 °C water bath. During the digestion period, the mixture was stirred at intervals of 15 min.

After the reaction was completed, standard medium—Dulbecco’s modified Eagle’s medium (Wako Pure Chemical Corp.) (20mL) containing 10% fetal bovine serum (Nichirei Biosciences Inc., Tokyo, Japan) and 1% penicillin-streptomycin solution (Wako Pure Chemical Corp.)—was immediately added to buffer the collagenase activity before filtering the resultant solution. The filtrate yields were centrifuged at 170 *× g* for 5 min at 25 °C and the supernatant was carefully removed. The resulting stromal vascular fraction was cultured in standard medium in an incubator at 37 °C for 3 h. The culture dishes were then washed thrice with PBS to remove the unattached cells. Only ADSCs were considered to remain on the bottom surface, which were then cultured with the standard medium and subcultured once 80% confluency was achieved.

***Local tissue scoring***

Modified Rissing scoring wes based on (0) no visible evidence of infection; (1) minimal erythema without bone destruction, without abscess; (2) erythema with bone formation and minimal bone destruction; (3) abscess with new bone formation, bone destruction, and purulent exudates; and (4) severe bone resorption, abscess, and total femur involvement [2, 3]

***Real-Time reverse-transcription polymerase chain reaction (RT-PCR)***

At the established endpoint (POD14), total RNA was extracted from the knee tissue of the rats (n = 6 rats per group) using a NucleoSpin RNA II kit (Takara Bio, Otsu, Japan) according to the manufacturer’s instructions. The absorbance of the total RNA was determined using an ultraviolet-visible spectrophotometer at 260/280 nm (NanoDrop Lite; Thermo Fisher Scientific). For real-time RT-PCR, mRNA (6 μg) was reverse-transcribed using a Revert Aid First-Strand complementary deoxyribonucleic acid Synthesis Kit (Thermo Fisher Scientific) and thermal cycler (T100TM Thermal Cycler; Bio-Rad, Hercules, CA, USA). Real-time PCR assays were conducted on ABI Prism 7900 apparatus (Applied Biosystems, Foster City, CA, USA) using SYBR Green PCR Master Mix (Applied Biosystems) according to the manufacturer’s instructions under the following thermal cycling profile: 40 cycles of 95 °C for 30 s, 55 °C for 60 s, and 72 °C for 60 s; 95 °C for 60 s; and 55°C for 30 s. Primers were purchased from Hokkaido System Science Corp., Ltd. (Hokkaido, Japan) (Supplementary Table S1). All values were normalized to the level of the GAPDH gene, and relative gene expression levels were calculated using the 2-ΔΔCt method [4].

**Reference**

1. Hamada, T., Matsubara, H., Yoshida, Y., Ugaji, S., Nomura, I., Tsuchiya, H. Autologous adipose-derived stem cell transplantation enhances healing of wound with exposed bone in a rat model. *PLOS ONE.* **14**, e0214106 (2019).

2. Rissing, J.P., Buxton, T.B., Weinstein, R.S., Shockley, R.K. Model of experimental chronic osteomyelitis in rats. *Infect. Immun.* **47**, 581‒586 (1985).

3. Shandley, S., Matthews, K.P., Cox, J., Romano, D., Abplanalp, A., Kalns, J. Hyperbaric oxygen therapy in a mouse model of implant‐associated osteomyelitis. *J. Orthop. Res.* **30**, 203‒208 (2012).

4. Livak, K.J., Schmittgen, T.D. Analysis of relative gene expression data using real-time quantitative PCR and the 2- ΔΔCT method. *Methods.* **25**, 402‒408 (2001).

**Supplementary Table S1. Sequences of primers used in real-time reverse transcription-polymerase chain reaction**

| **Gene** | **Forward primer (5′–3′)** | **Reverse primer (5′–3′)** |
| --- | --- | --- |
| GAPDH | AGACAGCCGCATCTTCTTGT | CTTGCCGTGGGTAGAGTCAT |
| rCRAMP | TGTAGCAAGGCATCACAGCA | CTTTTCG GAGGAGTCCAGCC |
| TNF-a | ACAAGGCTGCCCCGACTAT | CTCCTGGTATGAAGTGGCAAATC |
| IL-6 | GTCAACTCCATCTGCCCTTCAG | GGCAGTGGCTGTCAACAACAT |
| IL-1b | CCTGTACGCCAACACAGTGC | ATACTCCIGCTTGCTGATCC |

GAPDH, glyceraldehyde 3-phosphate dehydrogenase; rCRAMP, rat cathelicidin-related antimicrobial peptide; TNF, tumor necrosis factor; IL, interleukin.
